# Association Between Internet Access and Human Papillomavirus (HPV) Vaccination Coverage in Brazilian Municipalities: An Ecological Study

**DOI:** 10.3390/vaccines14100863

**Published:** 2026-09-29

**Authors:** Letícia Martins Cândido, João Pedro Silvestre Armani, Livia de Lima Moura, Ana Lúcia Danielewicz, Eloisa Pavesi, Alberto Sumiya, Kátia Jakovljevic Pudla Wagner

**Affiliations:** 1 Graduate Program in Public Health, Federal University of Santa Catarina, Florianópolis 88040-900, SC, Brazil; leticia.mc@posgrad.ufsc.br (L.M.C.); ana.lucia.d@ufsc.br (A.L.D.); 2 Department of Biosciences and One Health, Federal University of Santa Catarina, Rodovia Ulysses Gaboardi, Km 3, Curitibanos 89520-000, SC, Brazil; joao.pedro.armani@grad.ufsc.br (J.P.S.A.); a.sumiya@ufsc.br (A.S.); 3 Maternity School of the University Hospital Complex, Federal University of Rio de Janeiro, Rio de Janeiro 22240-003, RJ, Brazil; liviadelimamoura@yahoo.com.br; 4 Graduate Program in Rehabilitation Sciences, Department of Physical Therapy, Federal University of Santa Catarina, Araranguá 88906-072, SC, Brazil; 5 Graduate Program in Neuroscience and Department of Morphological Sciences, Center of Biological Sciences, Federal University of Santa Catarina, Florianópolis 88040-900, SC, Brazil; e.pavesi@ufsc.br

**Keywords:** adolescent, papillomavirus vaccines, vaccination coverage, internet access, digital health, public health

## Abstract

**Background/Objectives:** Human Papillomavirus (HPV) vaccination is the primary strategy for preventing HPV-related cancers, yet vaccination coverage (VC) remains uneven worldwide. Internet access may affect vaccination adherence, but its relationship with HPV VC at the population level is poorly understood. Therefore, we aim to describe the distribution of HPV VC and its association with household internet access across Brazilian municipalities and macro-regions, by sex and year. **Methods:** This ecological study examined the association between household internet access and HPV VC among girls and boys aged 9 to 14 years in Brazilian municipalities between 2022 and 2024. Two analytical approaches were used: (1) Categorical: both variables were dichotomized by their national medians, and prevalence ratios (PR) were estimated by Poisson regression with robust variance; (2) Continuous: VC was modelled in percentage points (p.p.) and internet access in 10-percentage-point increments, using linear regression with robust variance, for all municipalities and stratified by the five Brazilian macro-regions. Models were adjusted for sociodemographic and contextual variables. **Results:** A total of 5560 municipalities were included. HPV VC was consistently higher among girls, with medians between 83.5% and 85.2% over the three-year period, than among boys, between 51.9% and 73.6%. The median proportion of internet access was 81.0%, and municipalities classified as having high internet access ranged from about 21.0% in the Northeast to 73.4% in the Central-West. In the categorical approach, municipalities with high internet access showed a higher prevalence of high VC only in 2024, among girls (PR 1.09, 95% CI 1.02–1.15) and boys (PR 1.08, 95% CI 1.02–1.15). In the continuous approach, each 10-p.p. increase in internet access was associated with an increase in VC of 0.59 p.p. among girls in 2023 (95% CI 0.12–1.06) and 0.81 p.p. in 2024 (95% CI 0.37–1.25), and of 0.76 p.p. among boys in 2022 (95% CI 0.19–1.33), 0.86 p.p. in 2023 (95% CI 0.25–1.46) and 1.04 p.p. in 2024 (95% CI 0.50–1.58). Stratified analyses showed positive associations in the North and South, and inverse associations among boys in the Southeast region of Brazil. **Conclusions:** Municipalities with higher internet access showed higher HPV VC, although the association varied across years, sex, and macro-regions. These findings highlight differences in HPV vaccination coverage according to municipal levels of household internet access.

## 1. Introduction

Human papillomavirus (HPV) is a sexually transmitted infection with which nearly all sexually active people will be infected at some point in their lives. It is usually symptomless, but it does pose a potential risk for different forms of cancer [1,2]. Chief among the strategies for preventing HPV infection is vaccination [3,4]. However, despite advances in cancer prevention through immunization, socioeconomic and geographic inequalities continue to constitute barriers to access to and uptake of vaccination [5]. One major inequality relates to information access [6], since knowledge about the vaccine—especially knowledge based on reliable data—is one of the main determinants of its acceptance and is essential for the success of immunization strategies [7,8].

Internet access has become a widely used information tool in the health sector, significantly impacting the real world, insofar as the type of content can influence decisions regarding HPV vaccination uptake [9]. In 2014, when the HPV vaccine was introduced by the Brazilian public health system, there was a significant increase in internet searches for the term “HPV vaccine,” and vaccination coverage for the first dose was high. In subsequent years, there was a gradual decline in both internet search volumes and vaccination coverage. In 2017, when the vaccine was first offered to boys, this trend was repeated [10,11]. These patterns concern how connected populations search for and engage with vaccine information, and not the availability of internet access itself.

The internet is now one of the primary sources of health information in society, making it an essential resource for encouraging preventive behaviors [12]. It can be used to spread reliable information about the HPV vaccine, strengthening health communication strategies and contributing to increased vaccination coverage [10,13]. Francis et al. [14], analyzing the impact of computer-, mobile device-, and internet-based interventions on the administration of HPV vaccine doses, concluded that there was evidence that interventions using communication technologies as their sole or primary mode for HPV vaccination intervention delivery could increase vaccination coverage. Such interventions, however, presuppose existing internet access and act upon it, so their effects cannot be extended to the mere availability of internet access in a territory.

Llavona-Ortiz et al. [12], in a study conducted in the United States in 2019 with social media users who were parents of adolescents, found that those who were exposed to information supporting the HPV vaccine were more likely to initiate vaccination for their children than those who had no exposure to such information. In contrast, those exposed to anti-vaccination content were more likely to delay or reject vaccination. Access to the internet therefore opens a channel through which both accurate information and misinformation circulate, and greater availability of health information does not in itself imply greater vaccine acceptance. Moreover, internet access is often intertwined with other socioeconomic and contextual determinants, several of which independently shape vaccination coverage [15]. Municipal internet access should therefore be examined as an indicator of the territorial conditions under which immunization programs operate, and not as an isolated determinant of vaccination adherence.

Despite advances in HPV vaccination, inequalities in internet access mirror the heterogeneity of vaccination coverage across regions of the world, suggesting a possible relationship between these phenomena [10]. Furthermore, this is a variable of recent interest in Brazil, where it was first investigated in the 2022 census, which found that 89.4% of the population lived in households with internet access, but that access was marked by geographical inequalities, ranging from 96.2% in the Federal District to figures below the national average in the North and Northeast regions [16]. These differences reflect socioeconomic inequalities that influence access to information, health services, and vaccination opportunities [10,13,14]. In this context, the availability of national data on internet access makes it possible to investigate, at the municipal level, its association with HPV vaccination coverage. Given the scarcity of studies on this relationship in Brazil, this study may inform the planning of public health communication policies and the promotion of vaccination in a more equitable manner.

Given the above, the objective of this study was to describe the distribution of HPV vaccination coverage and its association with household internet access across Brazilian municipalities and macro-regions among boys and girls aged 9 to 14 years between 2022 and 2024.

## 2. Materials and Methods

### 2.1. Study Design and Sample

This ecological study involved a sample consisting of all 5570 municipalities in Brazil. The data analyzed pertain to HPV vaccination coverage among children aged 9 to 14 years (both male and female), as this is the target age group for HPV vaccination in the Brazilian Ministry of Health’s National Immunization Program [3,17]. Vaccination coverage data were obtained from the National Health Data Network (Rede Nacional de Dados em Saúde, RNDS, Brazil) for the target years, namely, 2022, 2023, and 2024. Sociodemographic factors were analyzed using data from the 2022 demographic census published by the Brazilian Institute of Geography and Statistics (Instituto Brasileiro de Geografia e Estatística, IBGE, Brazil).

### 2.2. Outcome Variable

The study’s outcome variable was high HPV vaccination coverage. In Brazil, the vaccine is offered through the public health system to boys and girls aged 9 to 14 years. In 2024, the Ministry of Health changed its recommendation from two doses to a single dose, with the aim of expanding vaccination coverage [17]. Vaccination coverage data were obtained from the RNDS, accounting for doses of the quadrivalent, bivalent, and nonavalent HPV vaccines.

HPV vaccination coverage was estimated using the birth-cohort method adopted by the Brazilian Ministry of Health. The numerator included individuals with at least one recorded HPV vaccine dose accumulated over the eligible vaccination period according to birth cohort, rather than doses administered exclusively in the calendar year, while the denominator corresponded to the population aged 9–14 years in the year of coverage assessment. To ensure standardization across years, the definition used by the Ministry of Health since April 2024, which considers individuals with at least one recorded dose as vaccinated, was applied to 2022–2024 [3,4]. The retrospective application of this definition to 2022 and 2023 was based on the evidence supporting the single-dose schedule in adolescents aged 9 to 14 years, which underpinned the change in the national recommendation [17]. For each Brazilian municipality, vaccination coverage values were extracted and stratified by sex, single year of age (9 to 14 years), and reference year (2022, 2023, and 2024), according to the information provided in the official dashboard.

In some municipalities, vaccination coverage values exceeding 100% were identified, which may be related to the fixed population denominator across the three years and the expectation of progressive refinement of nominal records in the RNDS. In such cases, these values were truncated at 100%. The proportion of municipalities with coverage above 100% varied by sex, age and year, reaching a maximum of 55.8% among girls and 23.8% among boys.

Vaccination coverage was analyzed as both a continuous and a dichotomous variable based on sex- and year-specific national medians (values above the median classified as high coverage and those at or below it as low coverage). The use of a stratum-specific median, rather than a single cut-off based on the programmatic vaccination target, was motivated by the low coverage observed, particularly among boys, for whom most municipalities had below the recommended 90% target [17].

### 2.3. Exposure Variables

The exposure variable was household internet access in the municipalities, based on an affirmative response to the question “Does any resident have internet access at home?” collected in the 2022 demographic census. For each municipality, the proportion of households in which at least one resident had internet access was calculated, and the national median of these proportions was then determined. Based on this median, municipalities were classified into low access (below the median) and high access (equal to or above the median).

For the continuous analyses, the same municipal proportion of households with internet access was divided by 10, so that one unit of the variable corresponds to 10 percentage points (p.p.). Accordingly, the estimated coefficients express the change in vaccination coverage associated with each 10 p.p. increment in the proportion of connected households in the municipality. This transformation is linear and does not alter the statistical significance of the estimates, only the scale on which they are expressed, thereby facilitating interpretation, since coefficients per single p.p. would be of very small magnitude.

### 2.4. Adjustment Variables

The adjustment variables, derived from the 2022 Brazilian demographic census microdata, were organized into two categories: sociodemographic characteristics and contextual factors of the municipality.

#### 2.4.1. Sociodemographic Characteristics

Sociodemographic characteristics included the proportion of individuals in each category of the variables race and educational attainment per municipality [18]. Municipal racial composition was categorized according to the predominant racial group in the adult population as predominantly White or predominantly mixed-race, Black, or Indigenous. Black- and Indigenous-predominant municipalities were included in the latter category because of their small numbers (*n* = 9 and *n* = 29, respectively), allowing all Brazilian municipalities to be retained while avoiding statistically unstable categories [19]. Educational attainment was defined as: no schooling to lower secondary education incomplete; lower secondary education complete to upper secondary education incomplete; and upper secondary education complete to higher education. The educational attainment and skin colour/race variables refer to the population aged 18 years or older, in accordance with the availability of municipal census aggregates.

#### 2.4.2. Contextual Factors

The contextual factors of interest were the indicators of socioeconomic, basic sanitation, and housing environment, derived from questions from the “household characteristics” section of the 2022 demographic census [20,21]. They were defined as the proportions of permanent private households in each municipality with access to piped water, with an exclusive-use bathroom, and with household waste collection. All three variables were retained on a continuous scale, expressed in percentage points (p.p.), thereby preserving the variability observed across municipalities and avoiding the imposition of cut-offs on continuous distributions.

### 2.5. Statistical Analysis

All statistical analyses were conducted using Stata 19.2. Significance was set at 5% (*p* < 0.05). Descriptive results were presented as medians and interquartile ranges (IQR). The choice of median-based measures and non-parametric tests was based on the observed skewness of the distributions, including the concentration of some municipal indicators near the boundaries of their scales. To compare the distribution of internet access between municipalities with low and high HPV vaccination coverage, the Mann–Whitney test was used separately by sex and year. Absolute differences in medians were also reported to describe the magnitude of the differences between the groups.

The association between internet access and vaccination coverage was estimated through two approaches.

In the first, both variables were dichotomized by their national medians, and prevalence ratios (PR) with 95% confidence intervals (CI) were estimated using Poisson regression with Huber–White robust variance.

In the second approach, vaccination coverage was modelled on a continuous scale, in p.p., by linear regression with robust variance, with internet access expressed in 10-p.p. increments. The β coefficients represent the difference, in p.p. of coverage, associated with each 10 p.p. increment in household internet access, holding the remaining covariates constant. Potential non-linearity in the exposure-outcome association was assessed using restricted cubic splines with four knots placed at the 5th, 35th, 65th and 95th percentiles of the exposure distribution, and the null hypothesis of linearity was tested by the F test. There was no evidence of departure from linearity in any of the six sex–year strata (corresponding to girls and boys in 2022, 2023, and 2024), with *p* values for non-linearity ranging from 0.129 to 0.999, and the linear specification was therefore retained.

In both approaches, three models were estimated per sex–year stratum: the crude model; Model 1, adjusted for educational attainment and skin colour/race; and Model 2, additionally adjusted for the proportions of households with access to piped water, with an exclusive-use bathroom, and with household waste collection. For each stratum, the percentage attenuation of the estimates between the crude and the adjusted models was calculated, quantifying the share of the crude association attributable to the covariates.

Regional heterogeneity in the municipality-level association was assessed using interaction terms between internet access and macro-region, which were jointly evaluated using F tests. Because these interactions were statistically significant in all six combinations of sex and year (F between 8.06–10.95; *p* < 0.001), separate municipality-level analyses were conducted within each of the five Brazilian macro-regions (North, Northeast, Southeast, South, and Central-West), using the same set of covariates included in Model 2.

The joint spatial distribution of the two indicators was examined using sex-specific municipal HPV vaccination coverage for the 2022–2024 period, summarized as the median of the annual coverage estimates for each municipality to provide a single measure for the study period. Vaccination coverage and internet access were each classified into three categories (3.0–<49.9%, 50.0–<69.9% and 70.0–100.0%), and bivariate choropleth maps were constructed separately for girls and boys by combining these categories, resulting in nine possible combinations. Maps were produced using the open-source Geographic Information System QGIS, version 3.34.3.

As sensitivity analyses, vaccination coverage was also dichotomized according to the programmatic threshold of 90%, corresponding to the target assumed by Brazil under the World Health Organization strategy (Table S1).

Multicollinearity among internet access and the covariates included in the adjusted models was assessed using variance inflation factors (VIF), estimated from auxiliary linear regressions using the same set of covariates. All values ranged from 1.07 to 2.32 (mean 1.56).

### 2.6. Ethical Considerations

Since this study was conducted using secondary, anonymized data in the public domain, there was no need for the study protocol to be reviewed by the research ethics committee, as permitted under Brazilian National Health Council resolution 510/2016.

## 3. Results

Of the 5570 Brazilian municipalities, 5560 were analyzed in the girls stratum and 5561 in the boys stratum, corresponding to 99.8% of the total. Missing information was restricted to access to piped water [8 municipalities: North (*n* = 1) and Northeast (*n* = 7)] and to aggregate vaccination coverage (2 municipalities in the girls stratum and 1 in the boys stratum). HPV vaccination coverage was consistently higher among girls, with medians of 83.5% (IQR 73.0–90.7) in 2022, 84.8% (IQR 76.0–91.3) in 2023, and 85.2% (IQR 77.1–91.7) in 2024. Among boys, the medians were substantially lower, but showed a marked upward trend over the period: 51.9% (IQR 40.7–63.7) in 2022, 68.0% (IQR 55.2–80.3) in 2023, and 73.6% (IQR 61.7–84.2) in 2024.

The median proportion of households with internet access was 81.0% (IQR 74.4–86.4). Regarding contextual indicators for municipalities, the medians were 75.8% (IQR 60.7–87.0) for access to piped water, 85.1% (IQR 69.3–94.8) for household waste collection, and 99.5% (IQR 96.2–99.9) for an exclusive-use bathroom. Most municipalities (83.6%) had populations whose level of educational attainment was predominantly lower, and in most municipalities (59.0%) the mixed-race, Black or Indigenous categories predominated.

Figure 1 shows the distribution of municipalities with high internet access by macro-regions. The proportion of municipalities with high internet access was substantially lower in the Northeast (21.0%, 95% CI 19.1–23.0) and North (31.1%, 95% CI 26.9–35.6) of the country, and considerably higher in the Central-West (73.4%, 95% CI 69.2–77.4), South (73.0%, 95% CI 70.3–75.5), and Southeast (63.3%, 95% CI 60.9–65.6).

The results for internet access according to low and high HPV vaccination coverage are presented in Table 1 (with IQRs) and illustrated in Figure 2. In all the years and for both sexes, the municipalities with high HPV vaccination coverage had statistically higher median internet access than the municipalities with low HPV vaccination coverage (*p* < 0.001). Overall, internet access was consistently higher in municipalities with high HPV vaccination coverage than in municipalities with low HPV vaccination coverage, with absolute differences ranging from 2.1 to 2.7 p.p. for girls and from 2.4 to 3.2 p.p. for boys (Table 1 and Figure 2).

Table 2 presents the results of the crude and adjusted PRs between internet access and high HPV vaccination coverage among 9- to 14-year-old boys and girls, from 2022 to 2024. Among girls, after adjustment for sociodemographic variables (Model 1), the prevalence of high vaccination coverage was 8% higher in municipalities with high internet access in 2022 (PR 1.08; 95% CI 1.02–1.14), 9% higher in 2023 (PR 1.09; 95% CI 1.03–1.15) and 13% higher in 2024 (PR 1.13; 95% CI 1.07–1.19), compared with municipalities with low internet access. Among boys, it was 8% higher in 2022 (PR 1.08; 95% CI 1.02–1.14), 10% higher in 2023 (PR 1.10; 95% CI 1.04–1.16) and 16% higher in 2024 (PR 1.16; 95% CI 1.09–1.22). After additional adjustment for contextual variables (Model 2), the association remained significant only in 2024, with a prevalence of high coverage 9% higher among girls (PR 1.09; 95% CI 1.02–1.15) and 8% higher among boys (PR 1.08; 95% CI 1.02–1.15).

The attenuation of the estimates between the crude model and Model 2 ranged from 63.3% to 106.6% among girls and from 70.0% to 100.0% among boys, being greatest in 2022 and progressively smaller in the following years. In 2022, the crude association was entirely absorbed by adjustment in both sexes, whereas in 2024 approximately one third of its original magnitude persisted after control for sociodemographic and contextual variables.

Table 3 presents the results of the analyses in which both vaccination coverage and internet access were modelled on a continuous scale. In the crude models, each 10 p.p. increment in household internet access was associated with increases of 1.87 to 2.13 p.p. in coverage among girls and of 3.10 to 3.77 p.p. among boys. After full adjustment, the estimates ranged from 0.37 to 1.04 p.p. and remained statistically significant in five of the six sex–year strata, the exception being girls in 2022.

The spatial distribution of median HPV vaccination coverage from 2022 to 2024 and internet access showed marked geographic heterogeneity across Brazilian municipalities (Figure 3). The maps revealed different combinations of the three categories of HPV vaccination coverage and internet access, with distinct geographic patterns between girls and boys. Municipalities in the highest vaccination coverage category were visually more frequent among girls than among boys. Substantial territorial variation was also observed in the joint distribution of internet access and HPV vaccination coverage.

The association between municipal internet access and the macro-region was presented in Table 4a,b. Among girls (Table 4a), positive and statistically significant associations were observed in the North and South regions. In the North, each 10 p.p. increment in internet access was associated with increases of 1.66 p.p. in coverage in 2023 (95% CI 0.07–3.24) and 1.47 p.p. in 2024 (95% CI 0.05–2.89), with no significant association in 2022. In the South, the association increased in magnitude over the period, from no association in 2022 to 0.99 p.p. in 2023 (95% CI 0.16–1.83) and 1.51 p.p. in 2024 (95% CI 0.72–2.31). In the Southeast, coefficients were negative in all three years, ranging from −0.83 to −0.89 p.p., without reaching statistical significance. No statistically significant associations were observed in the Northeast or the Central-West in any of the years analyzed.

Among boys (Table 4b), the pattern observed in the North and South regions was similar, although with a different temporal distribution. In the North, associations were significant in 2022 (1.75 p.p.; 95% CI 0.26–3.24) and 2023 (1.95 p.p.; 95% CI 0.21–3.70), with no significance in 2024. In the South, magnitude again increased over the period, reaching 1.33 p.p. in 2023 (95% CI 0.04–2.63) and 1.70 p.p. in 2024 (95% CI 0.52–2.88). In the Southeast, in contrast with the remaining regions, associations were negative and statistically significant in 2023 (−1.61 p.p.; 95% CI −2.91 to −0.31) and 2024 (−1.59 p.p.; 95% CI −2.76 to −0.42), indicating lower vaccination coverage among boys in municipalities with higher household internet access. As observed among girls, no significant associations were identified in the Northeast or the Central-West.

## 4. Discussion

HPV vaccination coverage was consistently higher among girls than among boys throughout the period, and household internet access was markedly unequal across the country, with the highest proportions of municipalities with high access in the Central-West, South and Southeast, and the lowest in the Northeast and North. Municipalities with higher internet access showed higher vaccination coverage, but this association was neither temporally nor territorially uniform, it was consistently observed in 2024 in both sexes, with less consistent findings in the preceding years, and was of greater magnitude among boys. Across macro-regions, the association was positive in the North and South, and inverse among boys in the Southeast. These findings indicate that the relationship between internet access and HPV vaccination coverage depends on sex and on the territorial context of Brazilian municipalities.

Although coverage remained higher among girls, the marked increase among boys from 2022 to 2024 should not be attributed to internet access alone. This temporal pattern may partly reflect the adoption of the single-dose schedule in 2024 and the later inclusion of boys in the national vaccination program in 2017, which provided less time for HPV vaccination practices to become established among the male population [4,5,8,9,17]. Moreover, there was inequality in the regional distribution of high internet access, with the highest proportions in the Central-West, South, and Southeast, and the lowest in the North and Northeast. Such regional inequalities—along with housing characteristics and levels of internet access, were also described by Nakayama et al. [15], based on data from 279,186 Brazilians. In addition to this regional disparity, Nakayama et al. [15] also found household location to be associated, with 88.5% of urban households enjoying access to the internet vis-a-vis just 53.2% of rural households.

Furthermore, the association between internet access and HPV vaccination coverage varied across Brazilian macro-regions, reinforcing the geographic heterogeneity previously reported for HPV vaccination in Brazil [22]. Positive associations were observed in the North in specific sex-year strata and in the South in 2024 for both sexes, whereas inverse associations were observed among boys in the Southeast in 2023 and 2024. These contrasting patterns suggest that connectivity alone may not translate uniformly into higher vaccination coverage and that its relevance may be shaped by the broader territorial context. In the North, where barriers to healthcare access remain substantial [23], internet connectivity may represent a broader marker of structural advantage and facilitate access to health information and communication. Conversely, in the more economically developed and highly connected South and Southeast [24], differences in internet access may be less indicative of favorable vaccination conditions, with healthcare organization, access to vaccination services, and locally adapted communication strategies potentially assuming greater relevance [25]. These findings support the need for regionally tailored digital vaccination strategies rather than assuming that greater internet availability has the same relevance across the country. Further studies incorporating regional aspects are needed to clarify this finding.

Hong et al. [26], in a study conducted in China among adult and older adult (≥45 years) populations, observed that internet access was strongly associated with neighborhood amenities (such as drinking water, bathrooms, and others) but not with health facilities or community organizations. Therefore, internet access can be considered a type of community resource; and since urban areas have broadband access, urban residents are more likely to be internet users [26]. Additionally, when analyzing the relationship between the digital divide and health disparities, Hong et al. [26] noted that self-reported health was significantly associated with internet use. Thus, although the internet is an easily accessible source of health information, it can also spawn inequalities in access to information [27]. In this context, it is essential to reduce the digital divide in healthcare and serve vulnerable populations, particularly those with low-income and ethnically diverse backgrounds, to promote equitable access, treatment, and health outcomes [28].

In our study, municipalities with high internet access had a higher prevalence of high HPV vaccination coverage than those with low internet access. A positive association was also observed by Sobral Filho et al. [10], who found a correlation between the volume of internet searches and vaccination coverage, indicating that media campaigns and health communication initiatives may contribute to public awareness and vaccination uptake. It should be noted, however, that such studies generally do not account for the socioeconomic conditions underlying internet access. In our study, the disappearance of the association after full adjustment in 2022 and 2023 suggests that internet access was closely intertwined with broader municipal socioeconomic and infrastructure conditions that may also influence access to preventive health services. The persistence of the association in 2024, despite the same adjustment structure, suggests that the relevance of internet access may vary across policy and vaccination contexts [29,30]. Consistent with this interpretation, Santos et al. [30], in their nationwide analysis of Brazilian adolescents, showed that HPV vaccine uptake is influenced by interconnected factors involving education, socioeconomic status, and access to healthcare. This could also be related to access to information through digital media, as engagement with health services and access to digital information sources were associated with higher HPV vaccination uptake [30]. In this regard, public-focused educational campaigns, combined with open dialogue between healthcare professionals and patients, may help demystify vaccines and reduce barriers to vaccination [29], while providing high-quality health information remains important for supporting informed health decisions [31].

In light of the above, digital interventions offer a promising way forward, potentially even addressing areas where traditional approaches fall short [6,11]. Different digital media have been used to convey health information to influence people’s behavior and, consequently, achieve better health outcomes; they include text message reminders, reminder alerts on electronic health records, educational videos, educational apps, and social media campaigns [6]. The growing use of social media platforms also represents an important dimension of HPV health education [32]. Platforms such as TikTok may facilitate the dissemination of accessible and engaging information about HPV prevention and vaccination, particularly among younger populations. However, the same platforms may also enable the rapid circulation of inaccurate or misleading health information, highlighting the importance of evidence-based digital health communication strategies. Recent research examining TikTok content related to HPV education further underscores the relevance of social media as an emerging environment for health information dissemination [32].

The strengths of this study include its nationwide scope, insofar as it analyzed all Brazilian municipalities for internet access, combined with the use of official and publicly accessible databases, lending its analyses national representativeness and reproducibility. Another key aspect is the three-year time period it covers, which lends its analyses a temporal dimension, capturing recent trends in HPV vaccination, complemented by analyses on a regional level, identifying areas with low internet access across the country. The study also employed complementary analytical strategies (dichotomous, continuous and stratified) as well as sensitivity analyses using programmatic thresholds and alternative handling of coverage values above 100%, which allowed the robustness of the findings to be assessed under different specifications. Based on the data presented, digital inclusion policies and digital strategies to promote HPV vaccination could help break down barriers to information and strengthen immunization efforts, particularly if they targeted areas where internet access is still limited. Internet access can also serve as an indicator of socioeconomic development and municipal infrastructure. Although the study does not directly measure digital intervention, the findings suggest that internet access inequalities should be considered when planning health communication strategies.

Our findings also have implications for the role of healthcare professionals in HPV vaccine communication. Healthcare professionals are trusted sources of vaccine information, and strong recommendations and effective patient-provider communication can support HPV vaccine uptake [33]. However, their ability to promote vaccination depends on adequate knowledge and communication skills, particularly for addressing concerns and vaccine hesitancy [34]. Moreover, evidence that healthcare professionals themselves may face practical and systemic barriers to accessing preventive health services highlights the importance of considering structural conditions alongside information and communication [35]. In this context, our findings add a territorial and digital perspective by showing that the association between internet access and HPV vaccination coverage varies across Brazilian regions. Digital channels may therefore support healthcare professionals in disseminating reliable vaccine information and reaching adolescents and families [33,34], but the regional heterogeneity identified in our study suggests that these strategies should be adapted to local connectivity and combined with face-to-face and outreach approaches in less connected municipalities.

This study has limitations. The ecological design prevents extrapolation to the individual level and precludes causal inference, and internet access may represent a broader socioeconomic context; only the availability of internet access was assessed, not its use to seek HPV information, so the association cannot be attributed to specific content. Educational attainment and skin colour/race were derived from the population aged 18 years or older and may not reflect households with adolescents, unlike the contextual indicators, measured at household level; grouping the mixed-race, Black and Indigenous categories precludes distinguishing patterns specific to each, although the few municipalities in which the latter two predominate make separate analysis unfeasible; and income, urbanization, population density and primary care supply were unavailable, so residual confounding cannot be excluded. Additionally, 2022 Census population data were used to construct the denominators for vaccination coverage estimates throughout the 2022–2024 period, as annual municipal population estimates disaggregated by single year of age and sex were unavailable. Consequently, changes in municipal population size and age structure during the study period may not be fully captured. Vaccination coverage estimates exceeding 100% were also observed in some municipalities, likely reflecting imprecision in the population denominators rather than in the vaccination records themselves. Furthermore, the transition to the single-dose regimen during the study period may have affected the comparability of the vaccination coverage data across the years.

Longitudinal and individual-level studies are nonetheless needed to determine whether internet access exerts an independent influence on HPV vaccination and to distinguish its potential informational effects from those related to socioeconomic determinants and access to health services.

## 5. Conclusions

Our study identified regional inequalities in internet access across Brazil, with higher proportions of municipalities with high access in the Central-West and South and lower proportions in the North and Northeast, alongside HPV vaccination coverage that was consistently higher among girls than among boys. At the municipal level, higher internet access was associated with higher vaccination coverage, although this association was observed consistently only in 2024 and was of greater magnitude among boys. Across macro-regions, the association was positive in the North and South and inverse among boys in the Southeast, indicating that its direction is not uniform across the country. These findings highlight the importance of considering inequalities in internet access and the territorial context when examining HPV vaccination coverage across Brazilian municipalities.

## Figures and Tables

**Figure 1:**
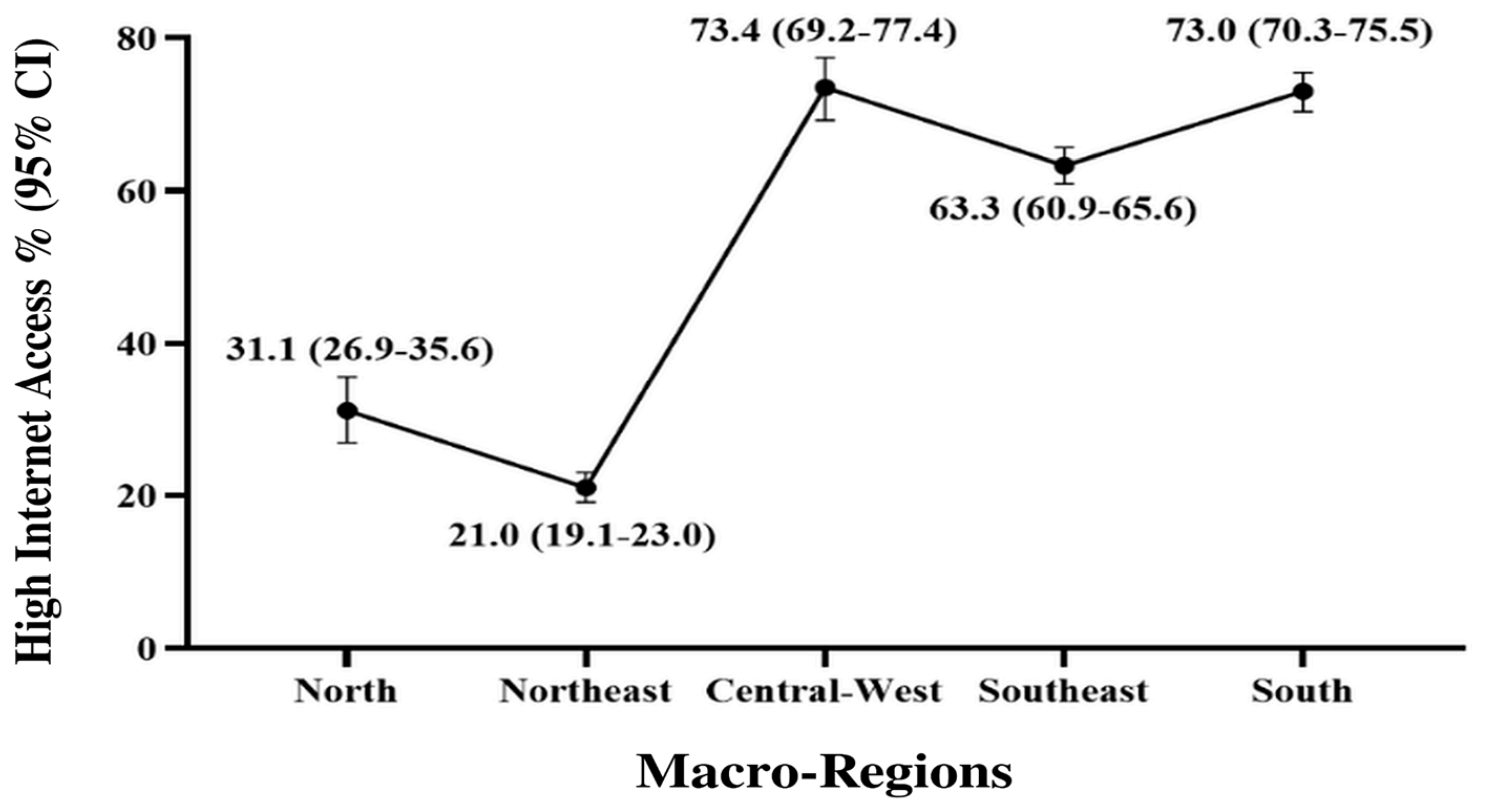
Proportion of municipalities with high internet access by macro-region. Brazil, 2022. Note: High internet access: proportion of households with internet access ≥ national median (81.0%). Number of municipalities by region: North (*n* = 450), Northeast (*n* = 1794), Central-West (*n* = 467), Southeast (*n* = 1668), South (*n* = 1191). Total: 5570.

**Figure 2:**
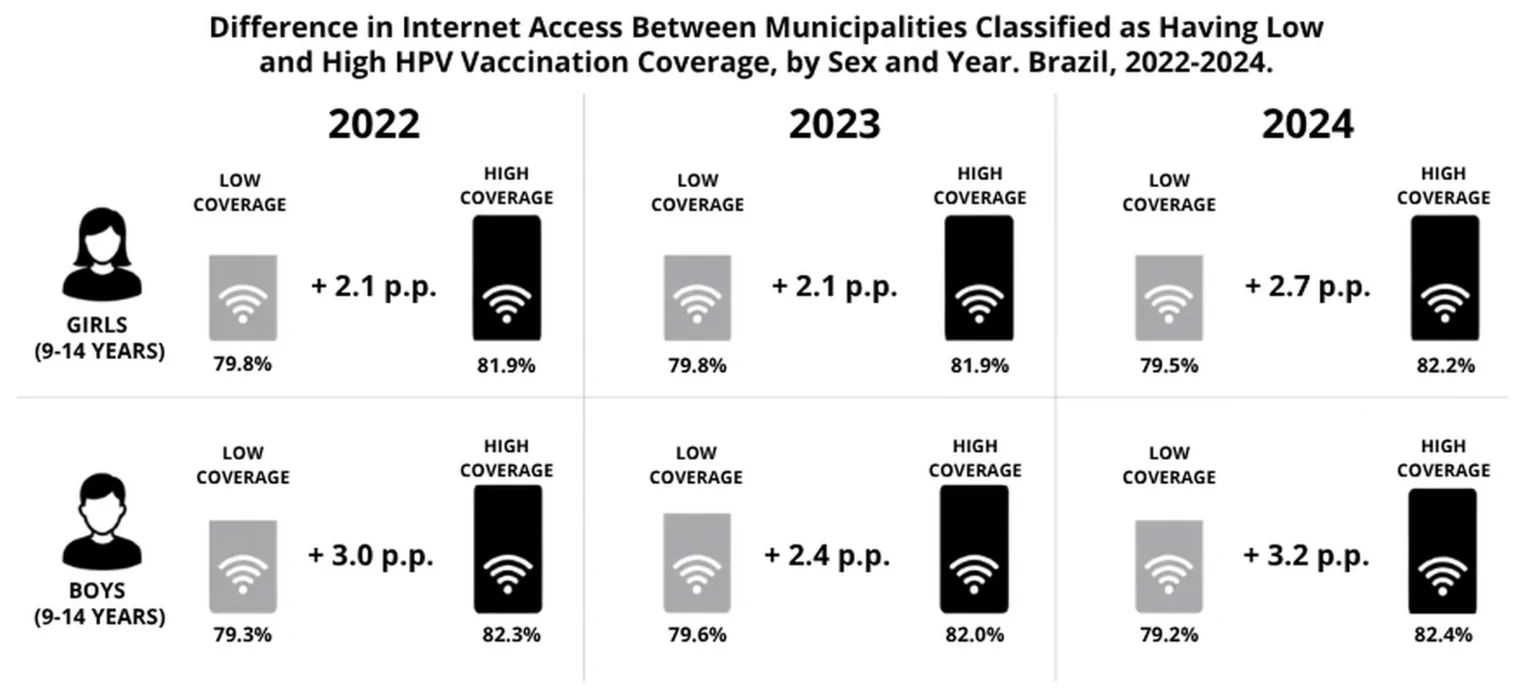
Internet access by HPV vaccination coverage, by sex and year. Brazil, 2022–2024. Note: The percentages represent the median proportion of households with internet access in the municipalities for each group. The values in percentage points (p.p.) correspond to the absolute difference between the high- and low-vaccination coverage groups (high–low). Vaccination coverage is dichotomized based on the median for each municipality by sex and year, using the following cutoff medians: girls in 2022 = 83.5%, 2023 = 84.8%, 2024 = 85.2%; boys in 2022 = 51.9%, 2023 = 68.0%, 2024 = 73.6%. The cutoff median for the proportion of households with internet access was 81.0%. In all years, the differences in internet access between the groups were statistically significant (Mann–Whitney test, *p* < 0.001).

**Figure 3:**
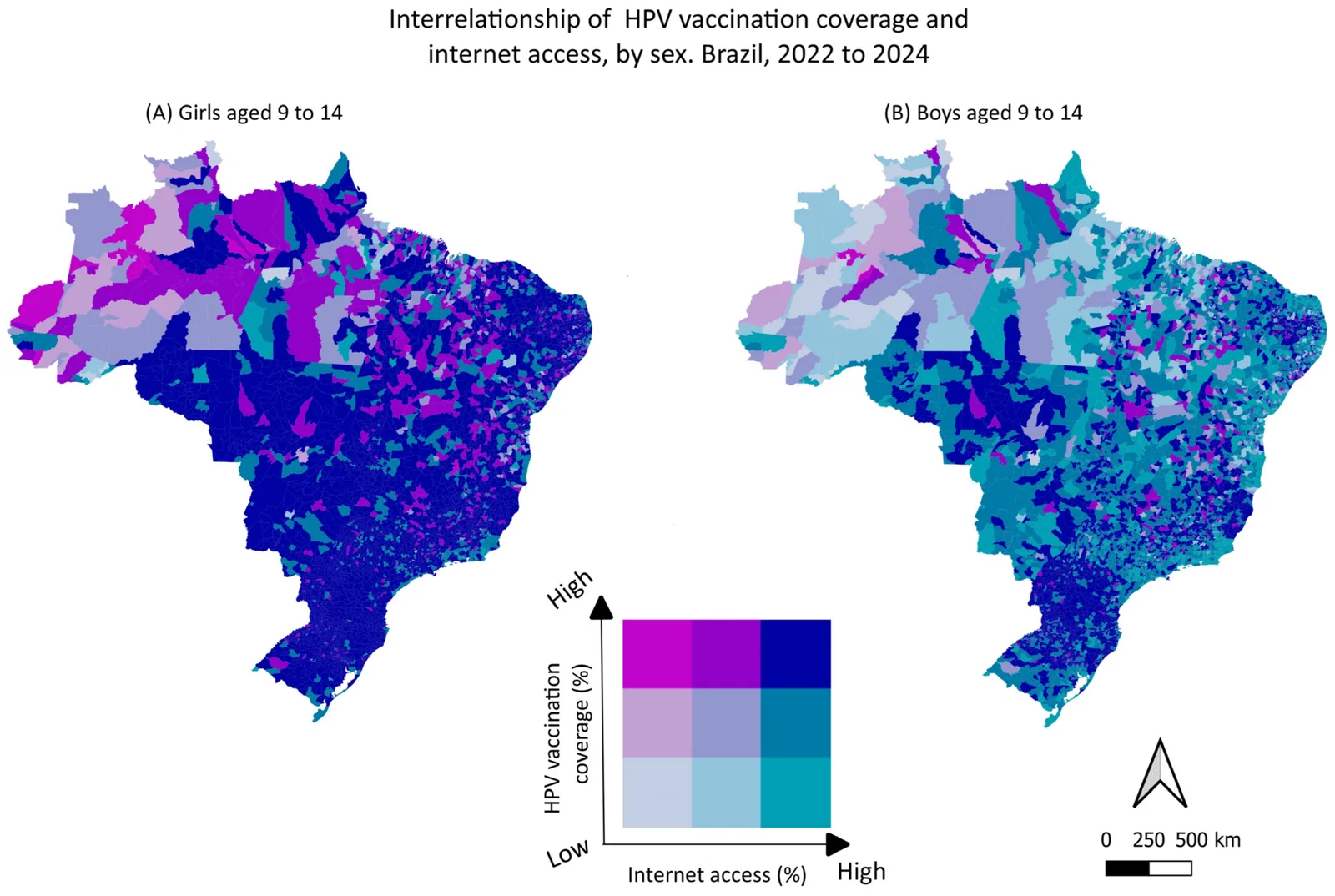
Spatial distribution of the interrelationship between HPV vaccination coverage and internet access across Brazilian municipalities, by sex, 2022–2024. Panel (**A**) represents girls aged 9–14 years and Panel (**B**) represents boys aged 9–14 years. HPV vaccination coverage represents the municipality-specific median of annual coverage estimates from 2022 to 2024. HPV vaccination coverage and internet access were classified into three categories (3.0–<49.9%, 50.0–<69.9%, and 70.0–100%), resulting in nine possible combinations in the bivariate color scale. HPV vaccination coverage increases along the vertical axis, whereas internet access increases along the horizontal axis. Gray represents municipalities in the lowest category for both HPV vaccination coverage and internet access, whereas dark blue represents municipalities in the highest category for both indicators. The remaining colors represent different combinations of the three categories of HPV vaccination coverage and internet access.

**Table 1:** Municipalities with internet access (median proportion of households) by low and high human papillomavirus (HPV) vaccination coverage among girls and boys aged 9 to 14, with absolute difference between groups, by sex and year. Brazil, 2022–2024.

Variables	HPV Vaccination Coverage								
	2022			2023			2024		
	Low	High	Δ	Low	High	Δ	Low	High	Δ
	Median (IQR)	Median (IQR)		Median (IQR)	Median (IQR)		Median (IQR)	Median (IQR)	
Girls									
Internet access †	79.8 (73.4–85.8)	81.9 (75.5–86.9)	+2.1	79.8 (73.4–85.8)	81.9 (75.5–87.0)	+2.1	79.5 (73.4–85.4)	82.2 (75.7–87.2)	+2.7
Boys									
Internet access †	79.3 (72.9–85.4)	82.3 (76.0–87.1)	+*3.0*	79.6 (73.2–85.7)	82.0 (75.8–87.0)	+2.4	79.2 (73.0–85.4)	82.4 (76.0–87.2)	+3.2

† Mann–Whitney test: *p* < 0.001 for all years and sexes. Δ = absolute difference (high–low coverage), in percentage points. IQR = interquartile range (25th and 75th percentiles).

**Table 2:** Prevalence ratio (PR) of the association between high internet access and high HPV vaccination coverage (above the sex- and year-specific national median) in municipalities among girls and boys aged 9 to 14 years, by sex and year. Brazil, 2022–2024.

Internet Access	2022				2023				2024			
	Crude PR (95% CI)	Adjusted 1 PR (95% CI)	Adjusted 2 PR (95% CI)	Attenuation (%)	Crude PR (95% CI)	Adjusted 1 PR (95% CI)	Adjusted 2 PR (95% CI)	Attenuation (%)	Crude PR (95% CI)	Adjusted 1 PR (95% CI)	Adjusted 2 PR (95% CI)	Attenuation (%)
Girls												
High internet access (vs. low internet access)	1.19 (1.13; 1.25)	1.08 (1.02; 1.14)	0.99 (0.93; 1.05)	106.6	1.19 (1.13; 1.26)	1.09 (1.03; 1.15)	1.04 (0.98; 1.10)	78.0	1.25 (1.19; 1.32)	1.13 (1.07; 1.19)	1.09 (1.02; 1.15)	63.3
Boys												
High internet access (vs. low internet access)	1.27 (1.20; 1.34)	1.08 (1.02; 1.14)	1.00 (0.94; 1.06)	100.0	1.23 (1.17; 1.30)	1.10 (1.04; 1.16)	1.04 (0.98; 1.10)	81.3	1.30 (1.24; 1.38)	1.16 (1.09; 1.22)	1.08 (1.02; 1.15)	70.0

PR = prevalence ratio, estimated by Poisson regression with robust variance; 95% CI = 95% confidence interval. High internet access: proportion of households with internet access ≥ national median (81.0%). High coverage: above the sex- and year-specific national median (girls: 83.5% in 2022, 84.8% in 2023, 85.2% in 2024; boys: 51.9%, 68.0% and 73.6%, respectively). Attenuation (%) = percentage reduction in the crude estimate in Model 2, computed on the log scale; values of 100% or above indicate that the crude association was fully accounted for by the covariates. Model 1: adjusted for education level and skin colour/race. Model 2: additionally adjusted for the proportion of households with access to piped water, with an exclusive-use bathroom, and with household waste collection, all modelled as continuous variables. Analyses were restricted to municipalities with complete data on all covariates: *n* = 5560 for girls and 5561 for boys.

**Table 3:** Difference in HPV vaccination coverage per 10-percentage-point increase in municipal household internet access, among girls and boys aged 9 to 14 years. Brazil, 2022–2024.

Internet Access	2022				2023				2024			
	Crude Model β	Model 1 β	Model 2 β	Attenuation (%)	Crude Model β	Model 1 β	Model 2 β	Attenuation (%)	Crude Model β	Model 1 β	Model 2 β	Attenuation (%)
Girls	2.12 (1.70; 2.53)	1.44 (1.00; 1.87)	0.37 (−0.14; 0.88)	82.4	1.87 (1.49; 2.25)	1.35 (0.94; 1.75)	0.59 (0.12; 1.06)	68.5	2.13 (1.78; 2.49)	1.54 (1.16; 1.92)	0.81 (0.37; 1.25)	61.9
Boys	3.77 (3.30; 4.23)	2.06 (1.58; 2.54)	0.76 (0.19; 1.33)	79.8	3.13 (2.64; 3.62)	1.92 (1.41; 2.43)	0.86 (0.25; 1.46)	72.6	3.10 (2.68; 3.53)	1.93 (1.48; 2.37)	1.04 (0.50; 1.58)	66.4

β = difference in percentage points of vaccination coverage per 10-percentage-point increase in the proportion of households with internet access, estimated by linear regression with robust variance; 95% CI = 95% confidence interval. Attenuation (%) = percentage reduction in the crude coefficient in Model 2. Model 1: adjusted for education level and skin colour/race. Model 2: additionally adjusted for the proportion of households with access to piped water, with an exclusive-use bathroom, and with household waste collection, all modelled as continuous variables. Analyses were restricted to municipalities with complete data on all covariates: *n* = 5560 for girls and 5561 for boys.

**Table 4:** (**a**) Difference in HPV vaccination coverage per 10-percentage-point increase in municipal household internet access, by macro-region and year, among girls aged 9 to 14 years. Brazil, 2022–2024. (**b**) Difference in HPV vaccination coverage per 10-percentage-point increase in municipal household internet access, by macro-region and year, among boys aged 9 to 14 years. Brazil, 2022–2024.

(**a**)							
**Macro-region**	***n***	**2022**		**2023**		**2024**	
		**Crude β (95% CI)**	**Adjusted β (95% CI)**	**Crude β (95% CI)**	**Adjusted β (95% CI)**	**Crude β (95% CI)**	**Adjusted β (95% CI)**
North	449	3.68 (2.64; 4.72)	1.68 (−0.00; 3.37)	3.53 (2.51; 4.55)	1.66 (0.07; 3.24) *	3.25 (2.28; 4.23)	1.47 (0.05; 2.89) *
Northeast	1787	0.75 (−0.10; 1.60)	0.12 (−0.81; 1.05)	0.32 (−0.45; 1.09)	−0.13 (−0.96; 0.70)	0.42 (−0.29; 1.12)	0.02 (−0.75; 0.79)
Southeast	1666	−2.67 (−3.41; −1.94)	−0.84 (−1.86; 0.18)	−1.98 (−2.65; −1.31)	−0.83 (−1.78; 0.11)	−1.67 (−2.31; −1.02)	−0.89 (−1.82; 0.03)
South	1191	0.08 (−0.69; 0.86)	0.40 (−0.52; 1.32)	0.57 (−0.14; 1.29)	0.99 (0.16; 1.83) *	0.99 (0.31; 1.67)	1.51 (0.72; 2.31) *
Central-West	467	1.22 (−0.73; 3.17)	1.24 (−0.99; 3.47)	0.86 (−1.01; 2.73)	0.91 (−1.22; 3.05)	0.64 (−1.08; 2.35)	0.63 (−1.33; 2.60)
(**b**)							
**Macro-region**	***n***	**2022**		**2023**		**2024**	
		**Crude β (95% CI)**	**Adjusted β (95% CI)**	**Crude β (95% CI)**	**Adjusted β (95% CI)**	**Crude β (95% CI)**	**Adjusted β (95% CI)**
North	449	3.85 (2.91; 4.79)	1.75 (0.26; 3.24) *	4.08 (2.93; 5.22)	1.95 (0.21; 3.70) *	3.08 (2.04; 4.11)	1.24 (−0.31; 2.80)
Northeast	1787	0.91 (0.06; 1.77)	0.22 (−0.73; 1.16)	0.56 (−0.39; 1.51)	0.12 (−0.93; 1.18)	0.31 (−0.51; 1.12)	0.07 (−0.86; 1.00)
Southeast	1667	−3.04 (−3.94; −2.14)	−0.86 (−2.15; 0.44)	−3.41 (−4.32; −2.50)	−1.61 (−2.91; −0.31) *	−2.78 (−3.57; −1.98)	−1.59 (−2.76; −0.42) *
South	1191	−0.15 (−1.37; 1.08)	0.39 (−1.03; 1.80)	0.64 (−0.47; 1.74)	1.33 (0.04; 2.63) *	1.12 (0.13; 2.11)	1.70 (0.52; 2.88) *
Central-West	467	1.03 (−0.86; 2.92)	1.41 (−0.80; 3.62)	1.27 (−0.92; 3.47)	1.75 (−0.80; 4.30)	1.36 (−0.71; 3.42)	1.81 (−0.52; 4.15)

(**a**) β = difference in percentage points of HPV vaccination coverage per 10-percentage-point increase in the proportion of households with internet access, estimated by linear regression with robust variance; 95% CI = 95% confidence interval. Adjusted models include education level, skin colour/race, and the proportions of households with access to piped water, with an exclusive-use bathroom, and with household waste collection. Estimates in bold and marked with an asterisk are statistically significant (*p* < 0.05). The internet and macro-region interaction term was statistically significant in all six sex–year strata (F from 8.06 to 10.95; *p* < 0.001). Analyses were restricted to municipalities with complete data on all covariates: *n* = 5560 for girls and 5561 for boys. (**b**) β = difference in percentage points of HPV vaccination coverage per 10-percentage-point increase in the proportion of households with internet access, estimated by linear regression with robust variance; 95% CI = 95% confidence interval. Adjusted models include education level and skin colour/race, and the proportions of households with access to piped water, with an exclusive-use bathroom, and with household waste collection, all modelled as continuous variables. Estimates in bold and marked with an asterisk are statistically significant (*p* < 0.05). The internet × macro-region interaction term was statistically significant in all six sex–year strata (F from 8.06 to 10.95; *p* < 0.001). Analyses were restricted to municipalities with complete data on all covariates.

## Data Availability

The data presented in this study were derived from resources available in the public domain, the National Health Data Network (Rede Nacional de Dados em Saúde, RNDS), available at https://infoms.saude.gov.br/extensions/SEIDIGI_DEMAS_VACINACAO_HPV/SEIDIGI_DEMAS_VACINACAO_HPV.html, accessed on 15 September 2026, and the 2022 Brazilian Demographic Census conducted by the Brazilian Institute of Geography and Statistics (Instituto Brasileiro de Geografia e Estatística, IBGE), available at https://sidra.ibge.gov.br/pesquisa/censo-demografico/demografico-2022/inicial, accessed on 15 September 2026.

## Supplementary Materials

The following supporting information can be downloaded at: https://www.mdpi.com/article/10.3390/vaccines14100863/s1, Table S1: Association between high municipal household internet access and the attainment of 90% HPV vaccination coverage among girls and boys aged 9 to 14 years. Brazil, 2022–2024.

## Abbreviations

CI Confidence interval; IQR Interquartile range; HPV Human papillomavirus; IBGE Brazilian Institute of Geography and Statistics; PR Prevalence ratio; RNDS National Health Data Network.
