# Supplementary material for: Association Between Internet Access and Human Papillomavirus (HPV) Vaccination Coverage in Brazilian Municipalities: An Ecological Study
Source: Vaccines (Basel). 2026 Sep 29;14(10):863. doi: 10.3390/vaccines14100863 (PMC13709507; doi:10.3390/vaccines14100863)
Supplement: Supplementary file 1 [file vaccines-14-00863-s001.zip › vaccines-4543273-supplementary.pdf]

vaccines

MDPI

Table S1. Association between high municipal household internet access and the attainment of 90% HPV vaccination coverage among girls and boys aged 9 to 14 years. Brazil, 2022-2024.

**Sex**	**2022**		**2023**		**2024**	
	**Municipalities ≥ 90% n (%)**	**Adjusted PR (95% CI)**	**Municipalities ≥ 90% n (%)**	**Adjusted PR (95% CI)**	**Municipalities ≥ 90% n (%)**	**Adjusted PR (95% CI)**
**Girls**	1,565 (28.1)	0.98 (0.89; 1.08)	1,652 (29.7)	1.07 (0.97; 1.17)	1,771 (31.9)	**1.17 (1.07; 1.28)***
**Boys**	92 (1.7)	1.05 (0.64; 1.73)	455 (8.2)	1.13 (0.92; 1.39)	685 (12.3)	**1.33 (1.12; 1.58)***

PR = prevalence ratio, estimated by Poisson regression with robust variance; 95% CI = 95% confidence interval. n (%) = number and proportion of municipalities attaining at least 90% coverage. High internet access: proportion of households with internet access ≥ national median (81.0%). Models adjusted for educational attainment and skin colour/race, and for the proportions of households with access to piped water, with an exclusive-use bathroom, and with household waste collection. Analyses were restricted to municipalities with complete data on all covariates (n = 5,560 for girls; n = 5,561 for boys). Estimates in bold and marked with an asterisk are statistically significant ($p < 0.05$). The 90% threshold corresponds to the target assumed by Brazil under the World Health Organization strategy.
